# Production of eicosapentaenoic acid by application of a delta-6 desaturase with the highest ALA catalytic activity in algae

**DOI:** 10.1186/s12934-018-0857-3

**Published:** 2018-01-13

**Authors:** Haisu Shi, Xue Luo, Rina Wu, Xiqing Yue

**Affiliations:** College of Food Science, Shenyang Agricultural University, Shenyang, 110866 People’s Republic of China

**Keywords:** Delta 6 fatty acid desaturase, Eicosapentaenoic acid, *Perilla* seed meal

## Abstract

**Abstract:**

*Dunaliella salina* is a unicellular green alga with a high α-linolenic acid (ALA) level, but a low eicosapentaenoic acid (EPA) level. In a previous analysis of the catalytic activity of delta 6 fatty acid desaturase (FADS6) from various species, FADS6 from *Thalassiosira pseudonana* (TpFADS6), a marine diatom, showed the highest catalytic activity for ALA. In this study, to enhance EPA production in *D. salina*, FADS6 from *D. salina* (DsFADS6) was identified, and substrate specificities for DsFADS6 and TpFADS6 were characterized. Furthermore, a plasmid harboring the *TpFADS6* gene was constructed and overexpressed in *D. salina*. Our results revealed that EPA production reached 21.3 ± 1.5 mg/L in *D. salina* transformants. To further increase EPA production, myoinositol (MI) was used as a growth-promoting agent; it increased the dry cell weight of *D. salina* transformants, and EPA production reached 91.3 ± 11.6 mg/L. The combination of 12% CO_2_ aeration with glucose/KNO_3_ in the medium improved EPA production to 192.9 ± 25.7 mg/L in the Ds-TpFADS6 transformant. We confirmed that the increase in ALA was optimal at 8 °C; the EPA percentage reached 41.12 ± 4.78%. The EPA yield was further increased to 554.3 ± 95.6 mg/L by supplementation with 4 g/L perilla seed meal (PeSM), 500 mg/L MI, and 12% CO_2_ aeration with glucose/KNO_3_ at varying temperatures. EPA production and the percentage of EPA in *D. salina* were 343.8-fold and 25-fold higher than those in wild-type *D. salina*, respectively.

**Importance:**

FADS6 from *Thalassiosira pseudonana*, which demonstrates high catalytic activity toward α-linolenic acid, was used to enhance EPA production by *Dunaliella salina*. Transformation of FADS6 from *Thalassiosira pseudonana* into *Dunaliella salina* with myoinositol, CO_2_, low temperatures, and perilla seed meal supplementation substantially increased EPA production in *Dunaliella salina* to 554.3 ± 95.6 mg/L. Accordingly, *D. salina* could be a potential alternative source of EPA and is suitable for its large-scale production.

**Electronic supplementary material:**

The online version of this article (10.1186/s12934-018-0857-3) contains supplementary material, which is available to authorized users.

## Background

Recent clinical and epidemiological studies have indicated that polyunsaturated fatty acids (PUFAs), such as eicosapentaenoic acid (EPA, 20:5^Δ5, 8, 11, 14, 17^), are essential nutrients and play crucial roles in the treatment of various human diseases [1–3], such as neuropsychiatric disorders [4], rheumatoid arthritis [5], inflammatory diseases [6, 7], hypertension [8],and cardiovascular diseases [9]. Currently, marine fish oil is the richest source of EPA; however, the depletion of global fisheries, high cost of EPA purification, and pollution of the marine environment [10] have prompted a search for alternative sources. Microorganisms, including microalgae, fungi, and bacteria, are the primary producers of EPA; microalgae are the most abundant source of EPA. Many recent studies have developed technologies to accumulate EPA directly from various microalgae [11].

**Fig. 1:**
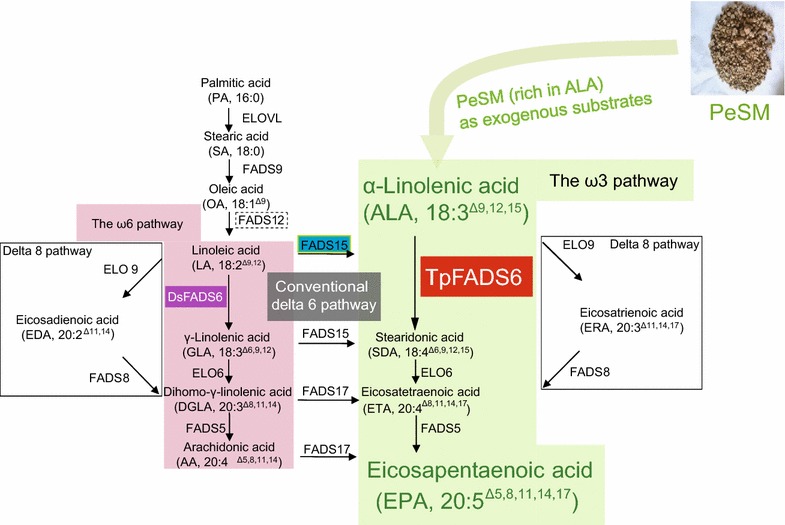
PUFAs biosynthetic pathways modified from Napier [44], where ALA-rich PeSM was added as exogenous substrate in medium to supply a sufficient source of ALA. The abbreviation of desaturases and elongases were described previously [34]

Among *Chlorophyceae*, *Dunaliella salina* (*D. salina*) is a unicellular wall-less alga that produces β-carotene and has a high tolerance to salt, temperature and light. In addition, this alga is quite easy to cultivate and has a relatively high lipid content, especially α-linolenic acid (ALA, 18:3^Δ9,12,15^). However, EPA production in *D. salina* is limited. Most algae produce EPA via the ω-3 pathway (Fig. 1); linoleic acid [LA, 18:2^Δ9,12^] forms a double bond between the carbon 15 and 16 from its carboxyl end to synthesize ALA, and EPA is produced by further desaturation and elongation of the carbon chain from ALA. In this pathway, the metabolic flux of *D. salina* PUFAs is blocked, maintaining ALA production. If this flux is unblocked, the EPA level in *D. salina* is expected to be enhanced. This can be achieved by the transformation of an exogenous ALA-preferring delta 6 fatty acid desaturase (*FADS6*) gene. Based on previous analysis of the substrate specificity of FADS6 from various species for LA and ALA [12], FADS6 of *Thalassiosira pseudonana* (TpFADS6), a marine diatom, has higher catalytic activity toward the ω3 substrate (ALA) than that of other algae, plants, and fungi. Its conversion efficiency is 68% in the ω-6 pool and 80% in the ω-3 pool [13], which is the highest ALA catalytic activity by far. Accordingly, the *TpFADS6* gene was transformed into *D. salina* in the present study.

Furthermore, we investigated various factors influencing EPA production in *D. salina.* Inositols are potentially valuable supplements for microalgal growth, especially myoinositol (MI) [14, 15]. Carbon sources provide energy necessary for algal growth. *D. salina* can use CO_2_ as a carbon source under photoautotrophic conditions [16]. *D. salina* is also capable of utilizing organic carbon sources, such as perilla seed meal (PeSM), which may promote its growth and/or EPA production [17]. PeSM solubility reaches 80% at pH 8.0, and it contains comprehensive essential amino acids and has a high ALA content; accordingly, it is a potential substrate for EPA production in *D. salina*.

In this study, we identified the *DsFADS6* gene from *D. salina* and characterized the substrate specificities for DsFADS6 and TpFADS6. Additionally, each FADS6 was overexpressed separately in *D. salina.* Furthermore, transgenic algae expressing each transgene were grown in Ben-Amotz medium to examine EPA production. Finally, we investigated the factors that influence EPA production by *D. salina*, including MI, CO_2_, low temperature, and PeSM supplementation.

## Results

### Identification of *DsFADS6* from *D. salina*

To identify genes encoding delta 6 desaturases involved in the biosynthesis of PUFAs in *D. salina*, a pair of degenerate primers was designed to target sequences corresponding to the heme binding motif of the cyt b5-like domain (HPGG) and the third His-rich motif (QIEHH) in *DsFADS6*. A 975-bp cDNA fragment from *D. salina* encoded a partial amino acid sequence containing a cyt b5-like domain in the N terminus and a His-rich motif in the C terminus; this region had high homology with delta 6 desaturases in other species.

To isolate the full-length cDNA clone, the insert was used as a probe to screen a cDNA library of *D. salina*. A full-length cDNA (*DsFADS6*) was identified by alignment and sequence analyses. The open reading frame of *DsFADS6* was 1329 bp and it encoded 442 amino acids.

**Fig. 2:**
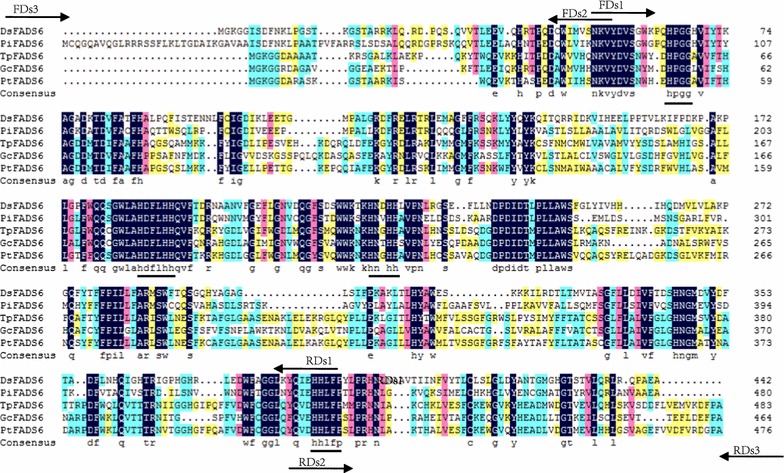
Multiple Sequence alignment of deduced amino acids of DsFADS6 with that of *Parietochloris incisa* (PiFADS6), *Thalassiosira pseudonana* (TpFADS6)*, Glossomastix chrysoplasta* (GcFADS6) *and Phaeodactylum tricornutum* (PtFADS6) (GenBank accession no. GU390532, AY817155, AAU11445 and AY082393) using DNAMAN. The three conserved HIS-rich motifs and a cyt b5-like domain are underlined. The arrows indicated the binding location of the degenerate primers used for amplification of partial sequence

A sequence alignment indicated that *DsFADS6* shares a similarity of 56.19% to *FADS6* from other taxa (Fig. 2). Homology was highest near the HPGG (the cyt b5-like) domain and in the three conserved HIS-rich domains (Fig. 2). These results suggest that *DsFADS6* may encode a delta 6 desaturase involved in the biosynthesis of γ-linolenic acid (GLA) and stearidonic acid (SDA) in *D. salina.*

### Characterization of the substrate specificity for DsFADS6 and TpFADS6

To characterize the substrate specificity of *DsFADS6* and *TpFADS6*, a 1329-bp fragment was amplified using FDs3 and RDs3 primers, and a 1455-bp fragment was amplified using FTp and RTp primers (primers listed in Additional file 1: Table S1). Successful expression of pYES2-DsFADS6 and pYES2-TpFADS6 was confirmed by western blotting in *Saccharomyces cerevisiae*. The fatty acid compositions are summarized in Table 1. When a single substrate was added, LA was catalyzed by DsFADS6 and the conversion rate was 24.3 ± 1.3%; however, ALA was not catalyzed by DsFADS6. When both substrates were added at the same time, the LA conversion rate by DsFADS6 was 23.8 ± 1.8%, and ALA was not catalyzed. These results show that DsFADS6 can catalyze LA conversion to GLA, but is not capable of catalyzing ALA.

**Table 1:** Fatty acid compositions (% w/w) of the total lipid contents of yeast transformants harboring the control plasmid (pYES2) and the recombinant plasmids (pYES2-DsFADS6 and pYES2-TpFADS6)

Transformants Fatty acid	pYES2 (control)			pYES2-DsFADS6			pYES2-TpFADS6		
	+LA	+ALA	+LA and +ALA	+LA	+ALA	+LA and +ALA	+LA	+ALA	+LA and +ALA
16:0 (PA)	22.9 ± 0.2	25.6 ± 0.2	20.5 ± 0.1	25.5 ± 0.4	21.9 ± 0.1	22.7 ± 0.3	23.4 ± 0.3	22.0 ± 0.6	24.0 ± 0.4
16:1 (PA)	12.7 ± 0.3	14.6 ± 0.2	12.9 ± 0.3	14.4 ± 0.2	16.3 ± 0.3	11.8 ± 0.2	14.6 ± 0.2	14.4 ± 0.4	13.1 ± 0.6
18:0 (SA)	11.4 ± 0.4	10.2 ± 0.6	11.8 ± 0.7	10.9 ± 0.4	12.5 ± 0.3	10.2 ± 0.4	12.0 ± 0.7	14.3 ± 0.5	9.3 ± 0.2
18:1 (OA)	12.7 ± 0.8	10.3 ± 0.7	11.6 ± 0.4	12.5 ± 0.2	11.8 ± 0.6	12.6 ± 0.4	11.3 ± 0.4	11.2 ± 0.3	10.3 ± 0.4
18:2 (LA, ω-6)	38.2 ± 0.3	ND	23.6 ± 0.1	29.6 ± 0.6	ND	16.1 ± 0.2	11.5 ± 0.5	ND	5.9 ± 0.6
18:3 (ALA, ω-3)	ND	39.6 ± 0.5	21.2 ± 0.6	ND	37.5 ± 0.2	21.6 ± 0.3	ND	4.3 ± 0.6	2.7 ± 0.2
18:3 (GLA, ω-6)	ND	ND	ND	7.2 ± 0.1	ND	5.0 ± 0.1	27.3 ± 0.3	ND	13.3 ± 0.3
18:4 (SDA, ω-3)	ND	ND	ND	ND	ND	ND	ND	34.9 ± 0.4	16.7 ± 0.2
LA conversion rate^a^	–	–	–	24.3 ± 1.3	–	23.8 ± 1.8	70.4 ± 0.2	–	69.3 ± 0.3
ALA conversion rate^a^	–	–	–	–	0	0	–	89.0 ± 2.2	86.1 ± 1.3

*ND* not detected

^a^Conversion rate = 100 × ([product]/[product + substrate])

When a single substrate was added, both substrates were catalyzed by TpFADS6 and their conversion rates were 70.4 ± 0.2 and 89.0 ± 2.2%, respectively. When both substrates were added simultaneously, the conversion rates of TpFADS6 for both substrates were similar to those observed when they were added individually, consistent with the results of Thierry et al. [13]. These results show that TpFADS6 is highly capable of catalyzing the conversion of both substrates to GLA and SDA. However, TpFADS6 did not exhibit substrate specificity.

Substrate concentration has no effect on catalytic activity in yeast expressing DsFADS6; similarly, we found that there was no substrate specificity for TpFADS6, irrespective of fatty acid concentration (data not shown).

### Overexpression of *TpFADS6* in *D. salina*

To construct expression plasmids, a 1329-bp fragment was amplified using pGEM-FDs/pGEM-RDs primers, and a 1455-bp fragment was amplified using pGEM-FTp/pGEM-RTp primers. Both fragments were ligated into the pGEM-CAT vector to obtain the pGEM-CAT-DsFADS6 and pGEM-CAT-TpFADS6 vectors (Additional file 2: Fig. S1a, b). The resulting pGEM-CAT-DsFADS6 and pGEM-CAT-TpFADS6 vectors were electrotransformed separately into *D. salina.* After transformation, small green colonies appeared at a frequency of 10–20 colonies/plate after 15 days. Ten pGEM-CAT-DsFADS6 transformants and 10 pGEM-CAT-TpFADS6 transformants were picked and cultured in liquid medium.

**Fig. 3:**
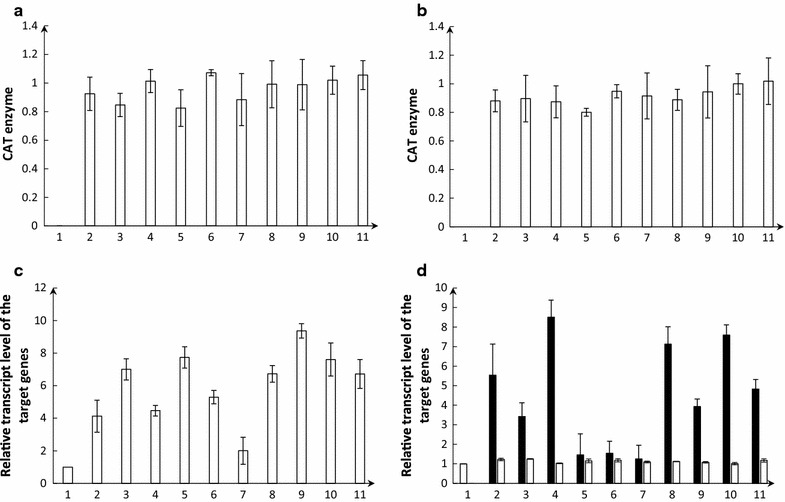
Analysis of CAT enzyme of pGEM-CAT-DsFADS6 transformants and pGEM-CAT-TpFADS6 transformants, respectively. Ten pGEM-CAT-DsFADS6 transformants and ten pGEM-CAT-TpFADS6 transformants were randomly picked out, and CAT enzymes were analyzed by ELISA method. Each column represented individual CAT protein of each transformants we picked out. **a** pGEM-CAT-DsFADS6 transformants; **b** pGEM-CAT-TpFADS6 transformants. Relative transcript level of DsFADS6, TpFADS6 in pGEM-CAT-DsFADS6 (**c**) and pGEM-CAT-TpFADS6 (**d**) transformants and the control strain (wild-type *D. salina*). The open bars represented the DsFADS6 transcript level and the black bars represented the TpFADS6 transcript level. Relative transcript level of the control strain was defined as 1

PCR amplification was used to identify *DsFADS6* and *TpFADS6* gene fragments in the transformants (Additional file 2: Fig. S1c, d). The target gene was detected in all transformants. Based on ELISA, CAT proteins were successfully expressed in all transformants and there were no significant differences in the expression level of *CAT* genes among *D. salina* transformants (Fig. 3a, b).

The expression levels of the *DsFADS6* and *TpFADS6* genes in all samples (including wild-type [WT] *D. salina*, pGEM-CAT-DsFADS6 transformants, and pGEM-CAT-TpFADS6 transformants) were analyzed after a 15-day cultivation period. Based on RT-qPCR, the transcript level of the *DsFADS6* gene in pGEM-CAT-DsFADS6 transformants was approximately two to ninefold higher than that in WT *D. salina* (Fig. 3c). The No. 9 pGEM-CAT-DsFADS6 transformant (Ds-DsFADS6-9) was used as a negative control; it had the highest transcript level of *DsFADS6* (9.4-fold higher than that in WT *D. salina*). There were significant differences in the transcript levels of *TpFADS6* among pGEM-CAT-TpFADS6 transformants. The No. 4 pGEM-CAT-TpFADS6 transformant (Ds-TpFADS6-4 transformant) had the highest *TpFADS6* transcript level (8.5-fold higher than that in WT *D. salina*), followed by the Nos. 8 and 10 pGEM-CAT-TpFADS6 transformants (7.1- and 7.6-fold higher than that in WT *D. salina*, respectively), and the transcript level was lower for the other pGEM-CAT-TpFADS6 transformants (Fig. 3d). The fatty acid profiles of Ds-TpFADS6-4, Ds-TpFADS6-8, and Ds-TpFADS6-10 were examined.

### Fatty acid profile of transformants

All transformants and WT *D. salina* were grown in Ben-Amotz medium at 26 °C for 30 days, and their fatty acid profiles were determined. There were no significant differences in dry cell weight, total fatty acid content, and C16 series levels among these transformants (Table 2). However, ALA levels were low in Ds-TpFADS6 transformants, in which the LA levels decreased to approximately 2 mg/L, and the EPA levels increased to 21.3 ± 1.5 mg/L, compared to 1.6 mg/L in WT *D. salina* (Table 2). The EPA content (expressed as a % of TFA) was 28.12 ± 5.75% in the Ds-TpFADS6-4 transformant, compared to 1.91 ± 0.21% in WT *D. salina.* (Table 2).

**Table 2:** Principal fatty acid profiles of each *D. salina* transformant

	DCW (mg/L)	TFA (mg/L)	C16 series (mg/L)^a^	LA (mg/L)	ALA (mg/L)	EPA (mg/L)	The percentage of EPA (TFA %)
WT *D. salina*	266.0 ± 30.7	78.6 ± 1.6	41.2 ± 1.5	5.3 ± 0.2	26.6 ± 4.7	1.6 ± 0.2	1.91 ± 0.21
Ds-DsFADS6-9	273.4 ± 22.0	73.2 ± 1.6	38.9 ± 2.6	5.4 ± 0.3	27.3 ± 2.5	1.5 ± 0.1	2.04 ± 0.14
Ds-TpFADS6-4	261.8 ± 18.2	74.1 ± 4.9	37.6 ± 1.9	2.0 ± 0.4	2.6 ± 0.6	20.7 ± 3	28.12 ± 5.75
Ds-TpFADS6-8	239.0 ± 33.5	79.9 ± 5.5	35.5 ± 2.6	2.1 ± 0.2	2.8 ± 0.2	21.3 ± 1.5	26.72 ± 2.26
Ds-TpFADS6-10	274.5 ± 19.4	83.8 ± 3.3	38.9 ± 2.1	2.2 ± 0.2	3.3 ± 0	19.1 ± 2.3	22.9 ± 3.65

^a^C16 series included 16:0, 16:1, 16:2, 16:3 and 16:4

### Biomass-enhancing effect of MI supplementation

Although TpFADS6 can promote the conversion of ALA to EPA in Ds-TpFADS6 transformants, the biomass of transformants was still low, limiting large-scale production. We attempted to resolve this problem by supplementing cultures with MI to stimulate the growth of transformants. Kichul et al. showed that supplementation with 500 mg/L MI had the greatest growth-promoting effect on *D. salina* (1.34 times higher growth than that of the control) [15].

**Fig. 4:**
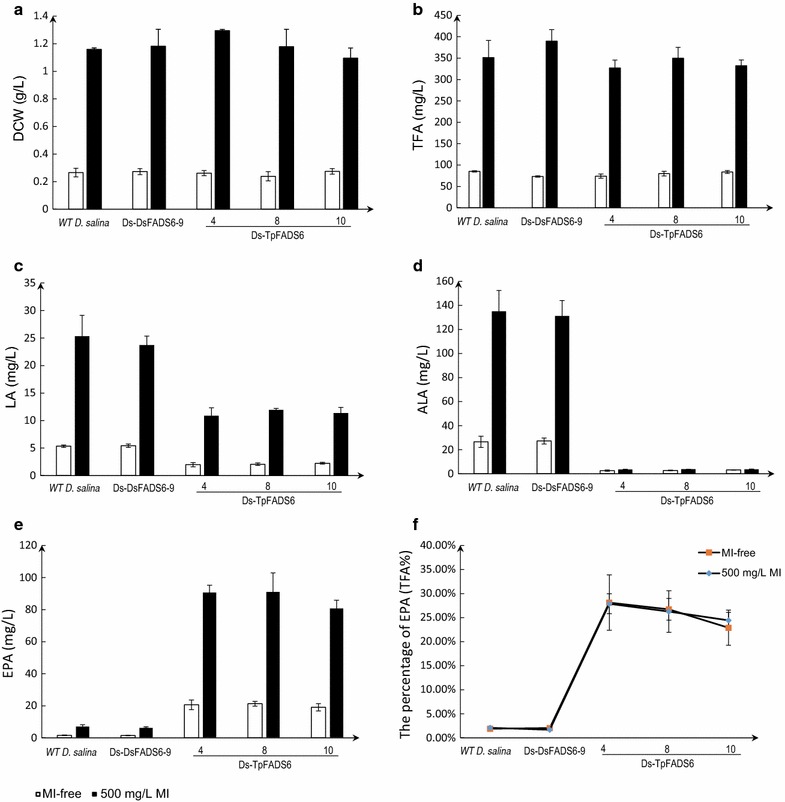
The effect of MI on each alga growth and principal fatty acid profiles. DCW (dry cell weight) (**a**), TFA (total fatty acids) (**b**), LA level (**c**), ALA level (**d**), EPA level (**e**) and the percentage of EPA (**f**) were determined in the control strain (WT *D. salina*) and transformants (including Ds-DsFADS6-9, Ds-DsFADS6-4, Ds-TpFADS6-8, and Ds-TpFADS6-10). Each alga was cultured in Ben-Amotz medium with 500 mg/L MI

The effects of MI on the growth of each transformant are summarized in Fig. 4. The dry cell weights (DCW) of WT *D. salina* and the transformants was increased significantly by supplementation with 500 mg/L MI, and reached sixfold higher than those of the MI-free control (Fig. 4a); TFA levels also increased (Fig. 4b). However, no significant changes were observed in the percentage of TFA (DCW %) among strains after the addition of MI. The levels of LA and ALA in Ds-TpFADS6 transformants was decreased by supplementation with MI, similar to the levels of the MI-free control (Fig. 4c, d). The EPA yield and percentage of EPA (TFA %) in the three Ds-TpFADS6 transformants were increased to 91.0 ± 4.2 mg/L (27.9 ± 2.1%), 91.3 ± 11.6 mg/L (26.3 ± 4.3%), and 81.1 ± 4.8 mg/L (24.4 ± 1.7%) by supplementation with 500 mg/L MI (Fig. 4e). However, there were no significant changes in the percentage of EPA (TFA %) between the MI group and the MI-free control (Fig. 4f).

### Lipid enhancement by supplementation with glucose/KNO_3_ and CO_2_

Glucose and CO_2_ in the culture medium can be used as carbon sources for algal growth, and their levels have significant effects on lipid accumulation in *D. salina* [16, 18]. We combined CO_2_ with glucose/KNO_3_ and examined DCW and lipid accumulation in *D. salina* transformants.

**Fig. 5:**
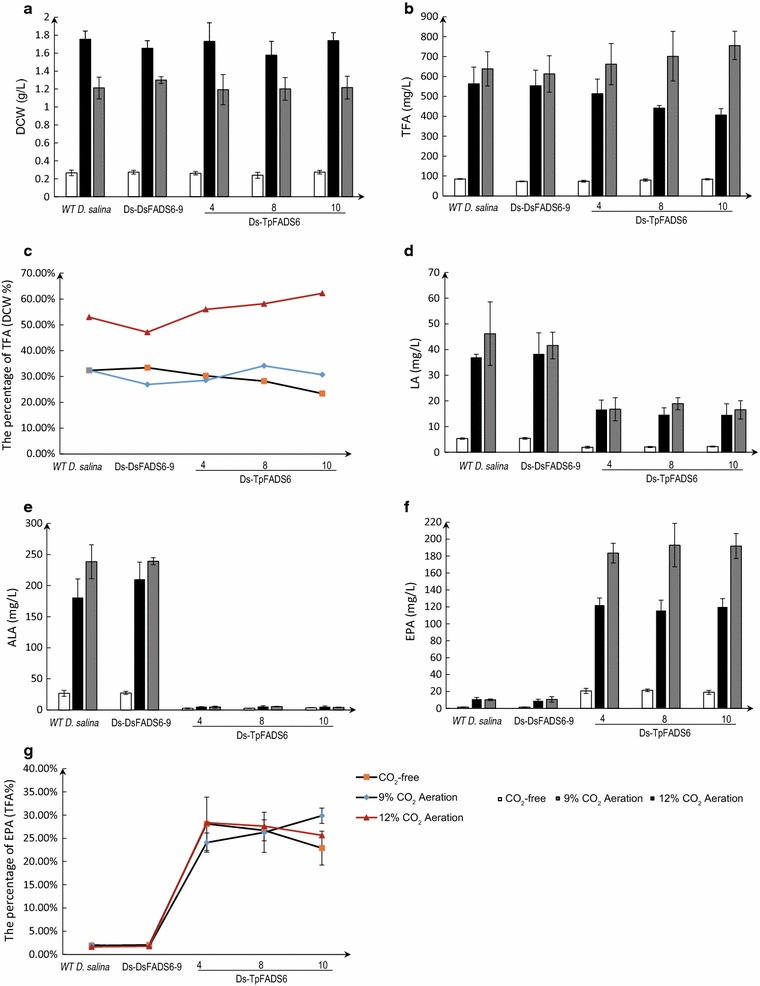
The effect of CO_2_ on each alga growth and principal fatty acid profiles. DCW (**a**), TFA (**b**), the percentage of TFA (DCW %) (**c**), LA level (**d**), ALA level (**e**), EPA level (**f**) and the percentage of EPA (TFA%) (**g**) were determined in the control strain (WT *D. salina*) and transformants (including Ds-DsFADS6-9, Ds-DsFADS6-4, Ds-TpFADS6-8, and Ds-TpFADS6-10). Each alga was cultured in Ben-Amotz medium with 500 mg/L MI and 9 or 12% CO_2_ aeration

Our results revealed that the DCW of WT *D. salina* and the transformants was increased significantly by aeration of 9% CO_2_, to approximately sevenfold higher than those of the control and approximately 50% greater than those of the MI group. The DCW of WT *D. salina* and all transformants was decreased by aeration with 12% CO_2_ (Fig. 5a). However, the TFA of all algae after aeration with 12% CO_2_ was greater than that observed after aeration with 9% CO_2_ (Fig. 5b). Based on the curve of the percentage of TFA (DCW  %), the TFA content in WT *D. salina* and all of the transformants was increased by approximately twofold by aeration with 12% CO_2_ compared to that with 9% CO_2_ and the control (Fig. 5c). The levels of LA and ALA in all algae exhibited similar patterns to those of MI-supplemented cultures (Fig. 5d, e). The Ds-TpFADS6 transformants presented noticeably higher EPA production with 9% and 12% CO_2_ aeration, reaching approximately 122.6 ± 7.8 and 192.9 ± 25.7 mg/L, respectively (Fig. 5f). However, there were no significant differences in the percentage of EPA (%TFA) (Fig. 5g). These results confirm that CO_2_ aeration in medium can enhance biomass and lipid production in *D. salina*; under 12% CO_2_ aeration, the level of EPA in Ds-TpFADS6 transformants increased by more than 19.2 fold relative to *D. salina*.

### Promoting the conversion of LA to ALA

**Fig. 6:**
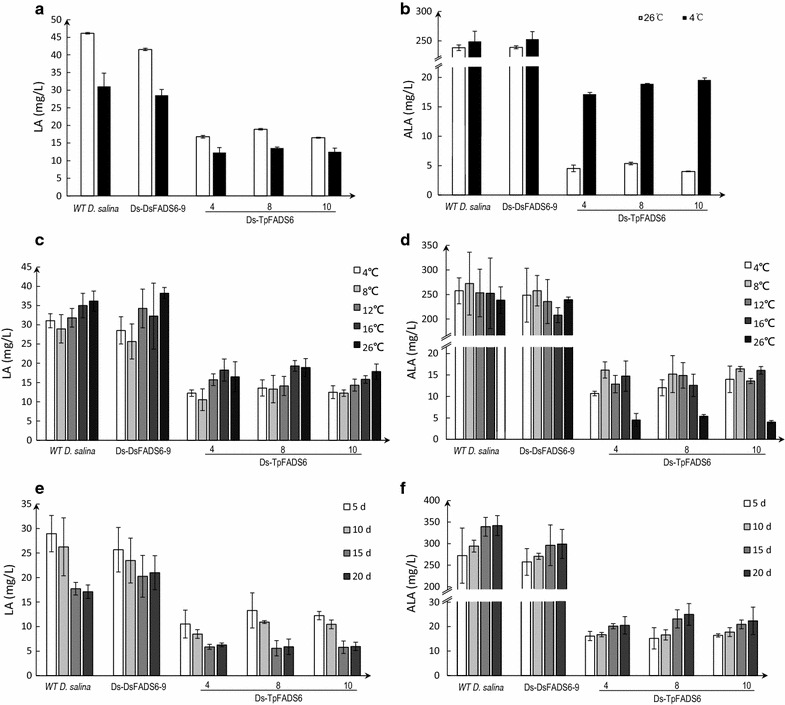
The effect of low temperature on promoting the conversion of LA to ALA. LA level (**a**) and ALA level (**b**) were determined in the control strain (WT *D. salina*) and transformants (including Ds-DsFADS6-9, Ds-DsFADS6-4, Ds-TpFADS6-8, and Ds-TpFADS6-10). Each alga was cultured in Ben-Amotz medium by supplementation with 500 mg/L MI, glucose/KNO_3_ and 12% CO_2_ aeration and conserved by 4 °C for 5 days. Optimization of culture temperature and time for each alga. LA level (**c**) and ALA level (**d**) were determined in the control strain (WT *D. salina*) and transformants (including Ds-DsFADS6-9, Ds-DsFADS6-4, Ds-TpFADS6-8, and Ds-TpFADS6-10) under 4, 8, 12, 16, and 26 °C (control) after 30-day cultivation for 5 days. After optimization of culture temperature, each transformant was cultured for another 5, 10, 15 or 20 days after 30-day growth under the optimal temperature. Each alga was cultured in Ben-Amotz medium by supplementation with 500 mg/L MI, glucose/KNO_3_ and 12% CO_2_ aeration. LA level (**e**) and ALA level (**f**) were determined in the control strain (WT *D. salina*) and transformants (including Ds-DsFADS6-9, Ds-DsFADS6-4, Ds-TpFADS6-8, and Ds-TpFADS6-10) under optimal temperature

The above results indicated that most ALA was converted to EPA, and only a small quantity remained in Ds-TpFADS6 transformants. We determined the fatty acid profile of transformants after they were cultured in Ben-Amotz medium by supplementation with glucose/KNO_3_ and 12% CO_2_ aeration at 4 °C for 5 days. Unexpectedly, ALA levels increased and LA levels decreased (Fig. 6a, b). A potential explanation for this result was that the DsFADS15 enzyme had greater catalytic activity at a low temperature, promoting the conversion of LA to ALA. However, the EPA levels did not change significantly, suggesting that the DsFADS6 enzyme had lower catalytic activity at a low temperature than at 26 °C. We examined a temperature gradient [4, 8, 12, 16, and 26 °C (control)] to determine the optimal conditions for ALA accumulation. The LA level at 8 °C was the lowest among the four temperatures 5 days after a 30-day cultivation period (Fig. 6c), and the ALA level at 8 °C was the highest among all temperatures (Fig. 6d). These results suggested that the DsFADS15 enzyme had the highest catalytic activity at 8 °C. After optimization of the culture temperature, each transformant was cultured for an additional 5, 10, 15, or 20 days after 30 days of growth at 8 °C. The LA level decreased and the ALA level increased as the cultivation progressed (Fig. 6e, f). The DCW, TFA, and EPA levels in this condition (at 8 °C and for another 20 days) did not exhibit significant changes (data not shown).

**Fig. 7:**
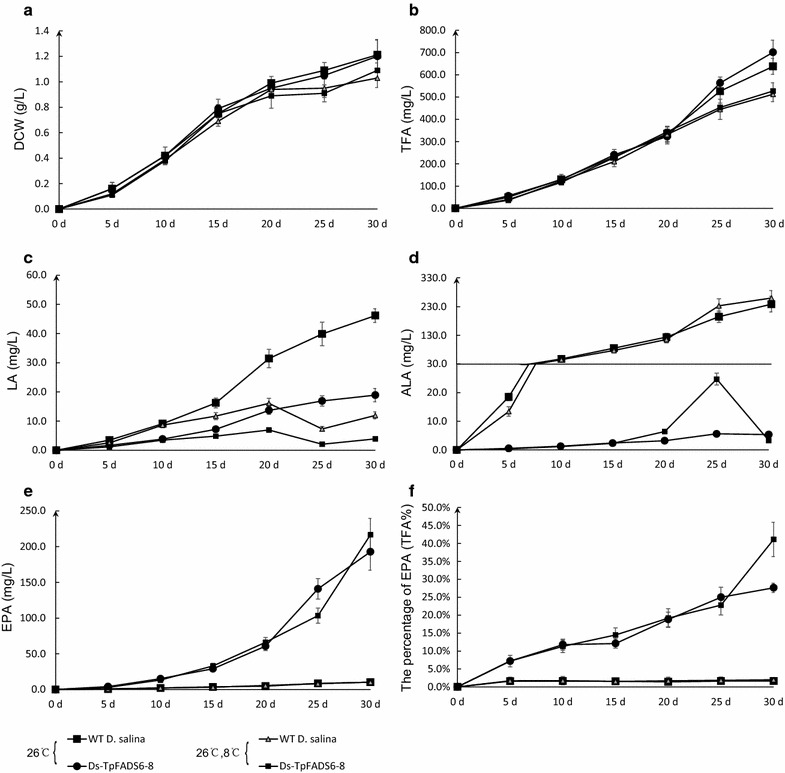
The growth curves of DCW (**a**), TFA (**b**), LA level (**c**), ALA level (**d**), EPA level (**e**) and the percentage of EPA (TFA %) (**f**) for WT *D. salina* and transformants (including Ds-DsFADS6-9, Ds-DsFADS6-4, Ds-TpFADS6-8, and Ds-TpFADS6-10) cultured in Ben-Amotz medium with 500 mg/L MI and 9 or 12% CO_2_ aeration under constant temperature (for 30-day cultivation at 26 °C) or under varying temperature (for 30-day cultivation at 26 °C but changing the temperature to 8 °C from 20 to 25 days)

By analyzing the growth curve for one of the Ds-TpFADS6 transformants (Ds-TpFADS6-8), we determined its DCW, TFA, ALA, and EPA levels for 30-day cultivation at 26 °C. There were no significant differences in DCW and TFA between two samples during the whole cultivation period (Fig. 7a, b), whereas the LA levels increased as the cultivation period progressed, with a greater increase from days 15 to 20 (Fig. 7c). The LA level showed a gradual increase.

To maximize the ALA level, we maintained the culture temperature at the optimal temperature (8 °C) from 20 to 25 days. The DCW and TFA levels at varying temperatures decreased slightly relative to those in a constant temperature (Fig. 7a, b). LA levels decreased sharply between 20 and 25 days, from 16.1 ± 1.7 to 7.3 ± 0.8 mg/L in WT *D. salina* (from 7.0 ± 0.8 to 2.1 ± 0.3 mg/L in the Ds-TpFADS6-8 transformant) (Fig. 7c). By contrast, ALA levels increased rapidly during this time period, from 115.6 ± 11.8 to 233.1 ± 24.1 mg/L in WT *D. salina* (from 6.4 ± 0.6 to 24.7 ± 2.1 mg/L in the Ds-TpFADS6-8 transformant) (Fig. 7d). We confirmed that the DsFADS15 enzyme presented high catalytic activity at 8 °C. The EPA level in the Ds-TpFADS6-8 transformant reached 216.8 ± 22.6 mg/L, which was only 24 mg/L higher than the yield obtained at a constant temperature (Fig. 7e). However, the percentage of EPA (TFA %) increased to 41.12 ± 4.78% at the end of cultivation, which was 1.5-fold higher than that observed at a constant temperature (27.64 ± 1.30%) (Fig. 7f).

### EPA enhancement by PeSM supplementation

**Fig. 8:**
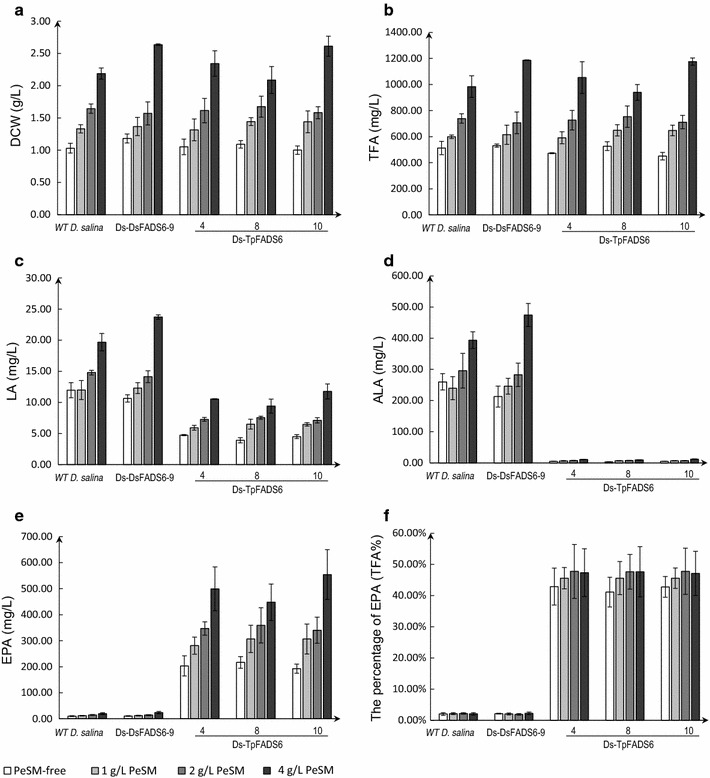
The effect of different concentrations of PeSM on each alga growth and principal fatty acid profiles. DCW (**a**), TFA (**b**), LA level (**c**), ALA level (**d**), EPA level (**e**) and the percentage of EPA (TFA %) (**f**) were determined in the control strain (WT *M. alpina*) and transformants (including Ds-DsFADS6-9, Ds-DsFADS6-4, Ds-TpFADS6-8, and Ds-TpFADS6-10). Each alga was cultured in Ben-Amotz medium by supplementation with 500 mg/L MI, glucose/KNO_3_ and 12% CO_2_ aeration under varying temperature, containing PeSM-free, 1, 2 or 4 g/L PeSM as the exogenous substrate

To further increase EPA production in *D. salina*, PeSM was added to the cultures. PeSM was composed of approximately 1% ALA (Additional file 1: Table S2). The DCW of WT *D. salina* and all of the transformants was increased significantly by supplementation with 1, 2, and 4 g/L PeSM, up to 2.6-fold (2.6 ± 0.2 g/L in Ds-TpFADS6-10 with 4 g/L PeSM) higher than that of the PeSM-free control (1.0 ± 0.1 g/L in Ds-TpFADS6-10) (Fig. 8a). The levels of TFA, LA, ALA, and EPA also increased (Fig. 8b–e). The EPA yield in the Ds-TpFADS6-10 transformant was increased to 306.5 ± 57.9, 340.2 ± 50.0, and 554.3 ± 95.6 mg/L by supplementation with 1, 2, and 4 g/L PeSM, respectively (Fig. 8e). LA levels were reduced by half, and only small amounts of ALA were detected. In addition, the Ds-TpFADS6-10 transformant accumulated EPA to a content of 47.81 ± 7.42% of the TFA after supplementation with 2 g/L PeSM (Fig. 8f, Additional file 3: Fig. S2).

## Discussion

Most algae synthesize VLC-PUFA via the FADS6 pathway (Fig. 1) [19]. Although *D. salina* is rich in ALA, the substrate specificity of DsFADS6 was unclear. We confirmed that it only catalyzed LA, and its conversion rate was low (24.3 ± 1.3%). In addition, TpFADS6 had no substrate specificity for the two substrates LA and ALA, but had a much higher ability to catalyze ALA and LA relative to that of FADS6 in *Micromonas pusilla* [12]. Therefore, we reasoned that transformation of the *TpFADS6* gene into *D. salina* may promote ω3-PUFA accumulation.

Cho et al. reported that exogenous genes can be easily introduced into *D. salina* [20]. We achieved the successful transformation of *D. salina* with the *DsFADS6* and *TpFADS6* genes using the electroporation method. However, the expression levels of the genes varied among transformants. This variation has two potential explanations: the *DsFADS6* or *TpFADS6* gene was randomly inserted into the *D. salina* genome at one or more sites, or the integration sites were random.

Both LA and ALA levels decreased in TpFADS6 transformants, and ALA levels decreased more substantially. In contrast, EPA levels and the percentage of EPA (TFA %) increased. The conversion rate of ALA to EPA in TpFADS6 transformants was greater than that in *S. cerevisiae*. This may be explained by the optimization of codon usage for TpFADS6 in *D. salina*, but not in *S. cerevisiae*. It may be also explained by differences in the levels of LA and ALA in *D. salina*. TpFADS6 had a higher conversion rate for ALA in *D. salina* (which had higher levels of ALA) than in *S. cerevisiae* (in which similar levels of both substrates were added).

Many previous studies have shown that auxins play key roles in algal growth [15, 21]. In this study, the DCWs of WT *D. salina* and the transformants were significantly increased by MI supplementation, which was expected, as MI is involved in many physiological functions [15, 22]. MI is safe for use in humans and its cost is relatively low, supporting its use as a growth-promoting agent for large-scale PUFA production in *D. salina*. Many studies have reported that CO_2_ fixation rates in microalgae are much greater than those in terrestrial plants [23]. Many microalgae tolerate CO_2_ levels of up to 12.0% [24], but algal growth can be affected by high CO_2_ levels [25]. Our results were consistent with these previous results. At a high CO_2_ level, the DCW of *D. salina* may be decreased, owing to the reduced levels of dissolved oxygen in the medium, and lipid biosynthesis in *D. salina* may increase. Aeration with CO_2_ in *D. salina* culture medium not only increases lipid levels but also promotes the conversion of CO_2_ (inorganic substance) to lipids (organic substance).

Surprisingly, after Ds-TpFADS6 transformants were maintained at 4 °C for 5 days, their ALA levels increased and LA levels decreased. We reasoned that the activity of the DsFADS15 enzyme was enhanced at the low temperature, promoting the conversion of LA to ALA and increasing the ALA level. These findings are similar to those of Okuda [26], who observed accumulation of EPA in *Mortierella alpina* at a low temperature (below 15 °C). However, FADS15 in *M. alpina* had high activity for C20 substrates, whereas DsFADS15 had high activity for C18 substrates. Ds-TpFADS6 transformants exhibited greater EPA accumulation under varying temperatures than under constant temperatures, suggesting that the LA-ALA-EPA pathway was more active under varying temperatures, and there was less LA flux through the ω6 pathway. Additional studies are needed to clarify the molecular mechanism of DsFADS15 activity under low temperatures.

Many plant seeds are rich in ALA, such as linseeds, tree peony seeds, sesame seeds, and perilla seeds [27–30]; among them, perilla seeds have the highest ALA content [29]. Accordingly, perilla seed oil has applications for both food and medicine. The byproduct of perilla seed oil processing (PeSM) is not widely used. Most PeSM is used as a protein source for animal feed [31]. In addition to ALA, PeSM also contains a high protein content and many other bioactive compounds, making it an inexpensive and undervalued algal material. *D. salina* is capable of using organic nitrogen for its growth [32, 33], and PeSM is a suitable nitrogen source. The solubility of PeSM is much greater than that of peony seed meal (PSM), which is used for *M. alpina* growth [34]. However, a high concentration of PeSM can still affect *D. salina* cell growth owing to increased levels of insoluble substances from PeSM in cultures under high concentrations.

EPA is found in a wide variety of marine microalgae. Recently, some remarkable findings in the generation of transgenic microalgae for enhanced EPA production have been reported [35–37]. *D. salina* is widely used for β-carotene production [38, 39]. In addition, it accumulates high levels of lipids and triacylglycerides. However, its EPA levels are low, and EPA production by *D. salina* has not been evaluated. In this study, several strategies were used to improve EPA accumulation by *D. salina*. However, investigations of EPA production by microalgae are still in the early stages, and an in-depth understanding of the factors that affect EPA production is still needed. Genetic engineering may be the most efficient means to improve EPA production in microalgae.

## Conclusions

In this study, we identified *FADS6* from *D. salina*. DsFADS6 had a preference for LA as a substrate and TpFADS6 had no substrate preference when expressed in *S. cerevisiae*. We successfully overexpressed the *TpFADS6* gene in *D. salina*; to our knowledge, this represents the first report of EPA bioproduction via the ω3 pathway in *D. salina.* EPA production in Ds-TpFADS6 transformants was enhanced to various degrees by MI, CO_2_, low temperature, and PeSM supplementation.

## Methods

### Strains and plasmids

*Dunaliella salina* (*D. salina*) was newly isolated from the reef on the beach in the northeast region of China. TpFADS6 gene (GenBank accession AY817155) was synthesized by Shanghai Sunny Biotechnology Co. Ltd; INVSc1 yeast strain (Invitrogen) was used for heterogeneous expression and substrate preference determination. Plasmid pGEM-CAT was used for FADS6 expression in *D. salina*.

### Media and cultural conditions

*D. salina* was grown at 26 °C on Ben-Amotz medium [40] in 12 h light (4500 lx)/12 h dark cycle. SC-U was synthetic minimal defined medium for *Saccharomyces cerevisiae* [12]. Biomass-enhancing medium was the Ben-Amotz modified medium by supplementation with 500 mg/L myo-inositol (MI) [15]. Lipid-producing medium was the Ben-Amotz modified medium by aerating with 12% CO_2_ level mixed with ambient air [16], and supplementation with 50 mM glucose (9 g/L) and 10 mM KNO_3_ (1 g/L) which was the result of our preliminary experiment for lipid production in *D. salina*. PeSM (1, 2 and 4 g/L) was added in lipid-producing medium as exogenous substrates for more lipid production, and was prepared by previous procedure for peony seed meal [34].

### RNA isolation and gene synthesis

1 μg RNA of *D. salina* cells was reverse-transcribed with QuantScript RT Kit according to the manufacturer’s instruction. The cDNA transcribed was used as a template for DsFADS6 amplification with primers. Codon-optimized TpFADS6 gene was synthesized by Biotechnology Co. Ltd and subcloned into the vector pUC57 and transformed into DH5α.

### Primer design, PCR amplification, and sequence analysis for DsFADS6 and TpFADS6

To identify genes encoding DsFADS6, a PCR-based cloning strategy was adopted. According to the available sequences information of FADS6 from *Parietochloris incisa*, *Thalassiosira pseudonana Glossomastix chrysoplasta and Phaeodactylum tricornutum* (GenBank accession nos. GU390532, AY817155, AAU11445 and AY082393), two highly degenerate primers (Additional file 1: Table S1) were designed to target sequences corresponding to the heme-binding motif of the cyt b5-like domain and the third His-rich motif in DsFADS6. The amplified product of expected length (DsFADS6 partial sequence, about 700 bp) was ligated into pMD19-T simple vector and then sequenced. DsFADS6 partial sequence obtained was used to do BLAST search on GenBank (NCBI). Two degenerate primers **(**Additional file 1: Table S1) were designed to clone the upstream sequence from HPGG and the downstream sequence from QIEHH. Both amplified products were sequenced and located the start codon and the stop codon. After the full length cDNA of DsFADS6 was amplified, it was then ligated into pMD19-T simple vector and sequenced.

For DsFADS6 and TpFADS6 gene amplification, primers were synthesized based on DsFADS6 and TpFADS6 gene sequences. The forward primers were FDs & FTp and the reverse primers were RDs and RTp (all primers are listed in Additional file 1: Table S1). PCR amplification, expression vector construction and sequencing were based on previous study [12], and recombinant plasmids were designated pYES2/NT C-DsFADS6 and pYES2/NT C-TpFADS6.

### Yeast transformation, heterologous expression in *S. cerevisiae*, and determination of substrate preference for DsFADS6 and TpFADS6

pYES2/NT C-DsFADS6 and pYES2/NT C-TpFADS6 were transformed into *S. cerevisiae* using the lithium acetate transformation method [41]. The selection procedure, SDS-PAGE gel and Western blotting for expression of DsFADS6 and TpFADS6 were analyzed as described previously [12]. After induction, cultures were supplemented with substrates (as described previously [12]). Substrate concentration experiment, lipids extraction and determination were also analyzed as described previously [34].

### Plasmid construction in *D. salina*

DsFADS6 and TpFADS6 genes were amplified with pGEM-FDs/pGEM-RDs and pGEM-FTp/pGEM-RTp primers, respectively, as shown in Additional file 1: Table S1. To construct an expression vector pGEM-CAT, the Ubiquitin-Ω (Ubil-Ω) promoter and nos terminator was amplified and cloned into the pGEM control vector to generate the expression vector pGEMΩ-CAT containing the Ubil-Ω promoter, nos terminator and CAT gene. DsFADS6 was digested and subcloned into *Hin*d III and *Xho* I sites to generate a plasmid designated pGEMΩ-CAT-DsFADS6 while TpFADS6 was subcloned into *Eco*R I and *Xho* I sites to generate a plasmid designated pGEMΩ-CAT-TpFADS6. The expression plasmids (pGEMΩ-CAT-DsFADS6 and pGEMΩ-CAT-TpFADS6) were transferred into *D. salina* cells according to the method described by Wang et al. [42].

### CAT assays and RT-qPCR analysis

The pre-procedure of CAT assays for stably *D. salina* transformants was followed by Wang’s description [42]. RT-qPCR analysis procedure were followed as previously described [34]. The transcript levels were calculated using the 2^−ΔΔ Ct^ method [43].

### PUFAs production for *D. salina* transformants

*D. salina* transformant cells in the liquid medium were grown for 30 days. The *D. salina* lysates obtained above were also used to determine dry cell weight (DCW) and fatty acid profiles in triplicates. FA profiles were investigated by using Gas Chromatography (GC) analysis as described previously [34]. *D. salina* transformant with the highest EPA production and the control were grown in biomass-enhancing medium for biomass enhancement, and grown in the lipid-producing medium for lipid enhancement. Finally, they were separately grown in EPA-enhancing medium for more EPA production.

## Additional files

**Additional file 1: Table S1.** Primers used in this study. **Table S2.** The composition of PeSM (%).

**Additional file 2: Figure S1.** Schematic illustration and transformation of expression vectors containing DsFADS6 gene and TpFADS6 gene, respectively. (a) Construction of vector pGEM-CAT-DsFADS6; (b) construction of vector pGEM-CAT-TpFADS6. Ten pGEM-CAT-DsFADS6 transformants and ten pGEM-CAT-TpFADS6 transformants were randomly picked out with PCR amplification for checking transformation of vector pGEM-CAT-DsFADS6 (c) and pGEM-CAT-TpFADS6 (d) into *D. salina* cells. *M* Marker; 1: positive control: DsFADS6 gene (c) and TpFADS6 gene (d); 2–11: each transformant.

**Additional file 3: Figure S2.** Gas chromatogram of fatty acids in WT *D. salina* with free-supplementation at 26 °C in Ben-Amotz medium (a) and Ds-TpFADS6-10 transformant by supplementing with 4 g/L perilla seed meal, 500 mg/L MI and 12% CO_2_ aeration with 50 mM glucose/10 mM KNO_3_ in varying temperature in Ben-Amotz medium (b).
