# Supplementary material for: Production of eicosapentaenoic acid by application of a delta-6 desaturase with the highest ALA catalytic activity in algae
Source: Microb Cell Fact. 2018 Jan 13;17:7. doi: 10.1186/s12934-018-0857-3 (PMC5766975; doi:10.1186/s12934-018-0857-3)
Supplement: Supplementary file 1 — Additional file 1: Table S1. Primers used in this study. Table S2. The composition of PeSM (%). [file 12934_2018_857_MOESM1_ESM.docx]

**Table S1** Primers used in this study

| Primer name | Restriction enzyme | Oligonucleotide sequence (5'-3')*^a^* | Function |
| --- | --- | --- | --- |
| FDs1 | - | C(A/C/T)(A/C)(G/A/C)AA(C/T)AA(G/A)GTITACGACGT | Amplification for the hemebinding motif of the cyt b5-like domain and the third His-rich motif (975 bp fragment) |
| RDs1 | - | T(T/G)(C/G)A(G/A)(C/T/G)CC(G/A)CC(T/G/A)(G/C)(T/A)GAACCA |  |
| FDs2 | - | GGCTTCCAGCCGCTGACGTC | Amplification for the upstream sequence from HPGG and the downstream sequence from QIEHH |
| RDs2 | - | AGCGGATTTTCTGAACCACC |  |
| FDs3 | *Hin*d III | CGCCCAAGCTTATGGGTAAAGGCGGAATTTC | DsFADS6 amplification for expression in *S. cerevisiae* |
| RDs3 | *Xho* I | CGGGGCCTCGAGTTAGGCCTCAGCGGGTTGAC |  |
| FTp | *Eco*R I | TACCGGAATTCATGGGAAAAGGAGGAGACGC | TpFADS6 amplification for expression in *S. cerevisiae* |
| RTp | *Xho* I | CGGGGCCTCGAGTTACATGGCAGGGAAATCCT |  |
| T7 | - | TAATACGACTCACTATAGGG | Target genes insert detection for yeast expression construction |
| pYES2.R | - | TCGGTTAGAGCGGATGTG |  |
| pGEM-FDs | *Hin*d III | CGCCCAAGCTTATGGGTAAAGGCGGAATTTC | DsFADS6 amplification for expression in *D. salina* |
| pGEM-RDs | *Xho* I | CGGGGCCTCGAGTTAGGCCTCAGCGGGTTGAC |  |
| pGEM-FTp | *Eco*R I | TACCGGAATTCATGGGAAAAGGAGGAGACGC | TpFADS6 amplification for expression in *D. salina* |
| pGEM-RTp | *Xho* I | CGGGGCCTCGAGTTACATGGCAGGGAAATCCT |  |
| RT-TpD6-F | - | ATGGGAAAAGGAGGAGACGC | qPCR for TpFADS6 transcript level measurement |
| RT-TpD6-R | - | AGGCATCGTCGGGGGTGATG |  |

*^a^* Underlined sequences indicate additional restriction sites.

**TABLE S2 The composition of PeSM (%)**

|  | crude protein (CP) | crude lipid (CL) | ALA | crude fiber (CF) | moisture (M) | ash (A) |
| --- | --- | --- | --- | --- | --- | --- |
| PeSM | 37.66 | 3.62 | 1.267 | 30.68 | 10.96 | 10.45 |
